# Analysis of Stability and Functionality of Coil and Piezoelectric Braille Modules Under Varying Temperature Conditions

**DOI:** 10.3390/mi16101112

**Published:** 2025-09-29

**Authors:** Krzysztof Zbroja, Anna Drabczyk, Oliwier Sobesto, Dominik Wojcieszczak, Mariusz Filipiec, Grzegorz Sapeta, Marcin Ostrowski, Patryk Kasza, Robert P. Socha

**Affiliations:** 1 CBRTP SA Research and Development Center of Technology for Industry, Zygmunta Modzelewskiego 77 St., 02-679 Warszawa, Poland; krzysztof.zbroja@cbrtp.pl (K.Z.); oliwier.sobesto@gmail.com (O.S.); dominik._.wojcieszczak@wp.pl (D.W.); filipiecm@gmail.com (M.F.); grzegorz.sapeta@cbrtp.pl (G.S.); marcin.ostrowski@cbrtp.pl (M.O.); patryk.kasza@cbrtp.pl (P.K.); 2 Department of Robotics and Mechatronics, AGH University of Krakow, Reymonta 13a St., 30-059 Kraków, Poland

**Keywords:** Braille module, electromagnetic coil, piezoelectric actuator, temperature stability

## Abstract

In this study, the performance and reliability of two different types of Braille modules, i.e., coil and piezoelectric, under varying temperature conditions were compared. The coil module works on the principle of electromagnetic forces generated by coils, while the piezoelectric module is based on the deformation of piezoelectric materials under electric voltage to move needles. The main purpose of this research was to discuss the stability and functionality of both modules within the temperature range from −30 °C to +50 °C. One thousand cycles of operation were conducted for each temperature step in 5 °C increments, focusing on the correctness of needle movement and system reliability. The results demonstrated that the piezoelectric module exhibited stable operation over the entire temperature range, while the coil module showed instabilities, such as self-jamming and overheating, above 20 °C. These problems were probably due to thermal expansion and reduced lubrication efficiency. These results underscore the piezoelectric module’s improved adaptation to high-temperature operation, making it a promising solution for applications requiring reliable operation under varying conditions.

## 1. Introduction

Braille modules serve as a fundamental component of assistive technologies for visually impaired individuals, enabling tactile reading through dynamically adjustable raised dots [1,2]. Over the years, various actuation mechanisms have been explored to optimize the performance of these modules, with electromagnetic coil-based [3] and piezoelectric-based solutions [4] emerging as dominant approaches. Each of these technologies presents distinct advantages and limitations, particularly in the context of stability and operational reliability under fluctuating environmental conditions.

Braille modules can be broadly classified into several types based on their actuation mechanism, including electromagnetic coil modules, piezoelectric modules, and microfluidic modules. Among these, coil and piezoelectric modules remain the most widely implemented due to their efficiency and reliability. Electromagnetic coil modules operate using solenoids that induce the movement of Braille pins through electromagnetic forces. When an electrical current flows through the coil, it generates a magnetic field that attracts or repels a ferromagnetic element connected to the pin, allowing it to extend or retract [5,6,7]. These modules are relatively simple in design, leveraging well-established electromagnetic principles, making them cost-effective for mass production. However, their high power consumption, susceptibility to thermal expansion, and increased friction and wear may result in jamming or operational failure over time [8,9]. Piezoelectric Braille modules, in contrast, utilize piezoelectric materials, such as lead zirconate titanate (PZT) and poly(vinylidene fluoride) (PVDF), which deform under an applied electric field to induce movement [10]. This actuation mechanism enables precise and rapid movement of Braille pins while maintaining energy efficiency [11,12]. These modules exhibit lower power consumption, a compact and lightweight design, and high mechanical precision, making them particularly advantageous for portable Braille displays.

In addition to their use in portable Braille displays, piezoelectric materials have been extensively investigated in a variety of actuator-based applications. Their ability to generate precise and rapid displacements under an applied electric field makes them particularly suitable for dynamic tactile interfaces, such as refreshable Braille pins and haptic feedback devices. For example, insect-scale aerial vehicles have successfully utilized piezoelectric actuation for ultrafast and high-frequency wing motion, demonstrating the capability of these materials to deliver reliable mechanical output even under demanding dynamic conditions [13]. Similarly, the milliDelta robot showcased millimeter-scale precision and high-bandwidth control achieved through piezoelectric actuation, proving its relevance for compact robotic systems where accuracy and responsiveness are essential [14]. Beyond robotics, ferroelectret nanogenerators were developed into thin, flexible patches that function as both self-powered loudspeakers and microphones, highlighting the dual role of piezoelectric-like materials in transducing electrical and mechanical energy for advanced wearable interfaces [15]. More recently, emerging research has emphasized their role in next-generation haptic devices and multifunctional tactile systems, where piezoelectric actuators ensure stable performance, scalability, and adaptability to diverse environmental conditions [16]. In addition, piezoelectric actuators play a central role in adaptive optics, where they allow real-time deformation of optical elements to correct aberrations, and in piezomotors, where nanoscale strain is converted into controlled macroscopic displacement for high-precision positioning systems. These examples illustrate the versatility of piezoelectric actuation in fields requiring stability, high resolution, and durability under diverse operating conditions.

Apart from actuation-oriented applications, piezoelectric materials are also widely utilized in energy-harvesting applications, such as Footwear Energy Harvesters (FEHs), which leverage mechanical deformation induced by walking to generate electrical energy [17,18]. FEH systems integrate piezoelectric materials into footwear soles, where they undergo cyclic mechanical deformation due to foot pressure and release. This deformation generates electrical energy, which can be harvested to power low-energy wearable devices, such as sensors, health monitoring systems, and wireless communication modules. The efficiency of these systems depends on factors such as the material properties of the piezoelectric layer, the applied mechanical force, and the frequency of foot movement. While FEH technology remains limited in terms of overall power output, ongoing advancements in material science and energy storage solutions, such as thin-film batteries and supercapacitors, are gradually improving their practical usability in real-world applications [19,20,21].

Nevertheless, despite their advantages, piezoelectric Braille modules involve higher fabrication costs due to the complexity of piezoelectric materials and the control circuitry required for precise actuation. In addition, although the control system for piezoelectric actuators is not overly complicated, traditional monolayer actuators often require relatively high operating voltages (around 100–200 V), which introduces challenges related to safety and insulation. For tactile and sensing applications, however, operating voltages below 50 V are usually sufficient, and the use of multilayer actuators can further reduce the driving voltage while maintaining the required displacement. These voltages may still exceed the range considered electrically safe, requiring careful design to ensure adequate sealing against moisture and to prevent accidental user contact and electric shock. Additionally, prolonged exposure to extreme environmental conditions may lead to gradual degradation of piezoelectric properties, potentially affecting long-term performance [4,22,23]. Importantly, temperature fluctuations can significantly affect the mechanical and electrical properties of actuation systems, leading to variations in performance, durability, and reliability. Electromagnetic coil modules may experience changes in resistivity, reduced lubrication efficiency, and expansion-induced jamming, while piezoelectric modules could be affected by alterations in material properties and voltage-dependent actuation behavior. Despite the importance of these factors, limited research has been conducted to systematically assess and compare the stability of these two Braille module types across a wide range of temperatures [24,25].

Apart from material and operational considerations, another crucial factor affecting the long-term reliability of Braille modules is temperature stability. Hence, the primary objective of this study was to evaluate the operational performance of electromagnetic coil and piezoelectric Braille modules under controlled temperature variations ranging from −30 °C to +50 °C. This research included subjecting each module to 1000 actuation cycles at each temperature increment of 5 °C. Assessment criteria included movement precision, system integrity, and failure mechanisms to determine the thermal resilience of each module type. The experimental setup included mounting modules on a specially designed frame within a climate chamber, simulating real-world operating conditions. This study also examined the effects of lubrication efficiency, thermal expansion, and electrical resistance variations, as these factors directly impact actuation speed, energy consumption, and long-term durability.

## 2. Materials and Methods

### 2.1. Materials

**Figure 1:**
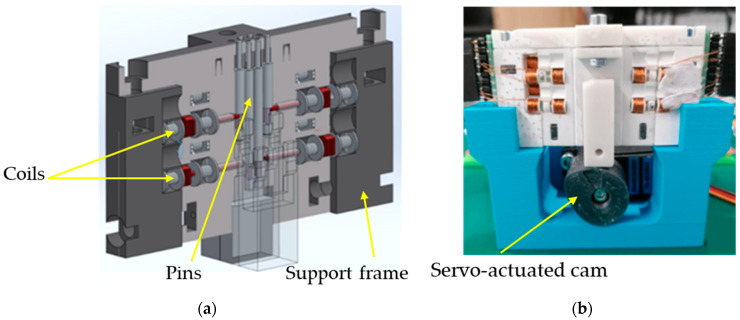
Electromagnetic coil Braille module presented as a schematic (**a**) and a photograph (**b**).

Two Braille modules with different operation mechanisms, i.e., a coil module and a piezoelectric one, were subjected to experiments. In the coil module, the movement of the needles needed to display Braille characters is controlled by a servo mechanism with a cam located beneath the panel. Electromagnetic coils act as locking mechanisms, holding the pins in position and preventing unintended movement. This module is presented below in Figure 1.

**Figure 2:**
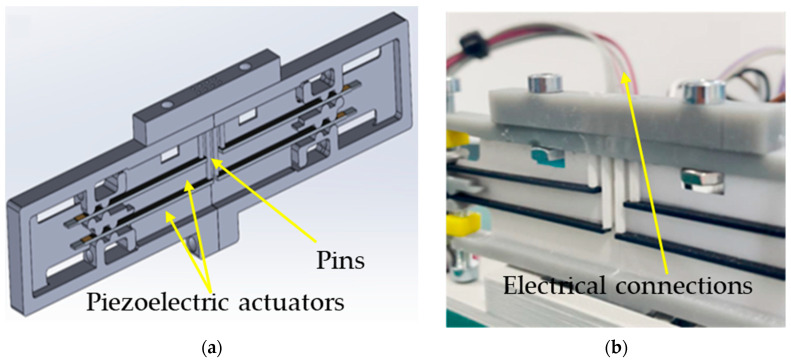
Piezoelectric Braille module presented as a schematic (**a**) and a photograph (**b**).

In the piezoelectric module, needle movement is induced using piezoelectric actuators that deform under the influence of an electric voltage, causing the needles to move. Its scheme and photography are demonstrated in Figure 2.

**Figure 3:**
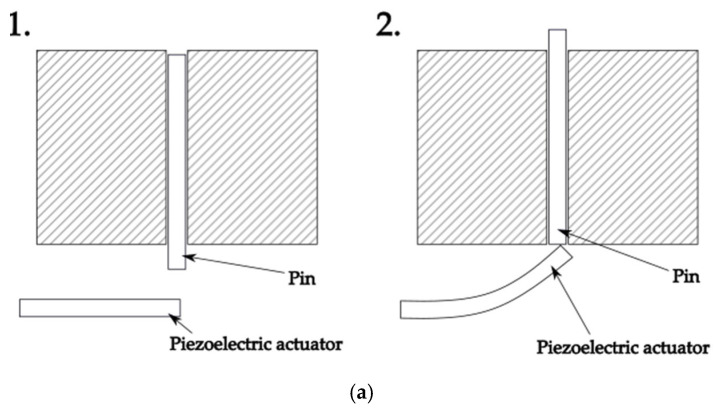

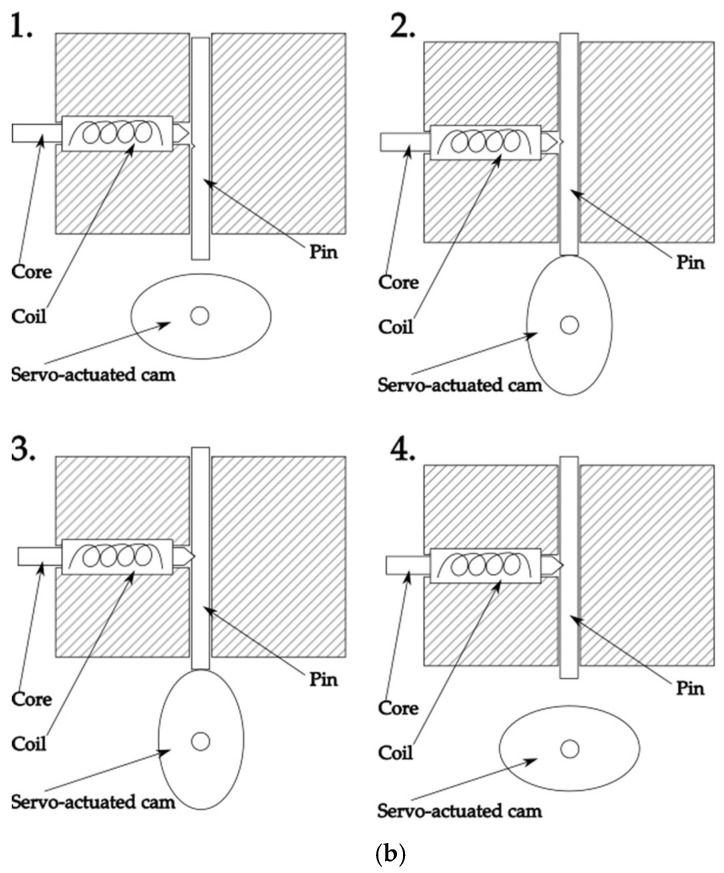
Schematic representations of the actuation principle of investigated Braille modules. (**a**) Piezoelectric module—(1) pin down, no voltage applied; (2) pin up, actuator deforms under applied voltage. (**b**) Coil module—(1) pin down, resting state; (2) servo initiates upward motion; (3) pin up, held in place by electromagnetic coil; (4) pin returns to down position after current is switched off.

Below, in Figure 3, the operation of both types of Braille modules is schematically illustrated. Figure 3a presents the piezoelectric module, while Figure 3b shows the coil-based module. These diagrams depict the sequence of pin motion between the resting (down) and active (up) positions.

In the piezoelectric module (Figure 3a), panel (1) shows the pin in the down position, when no voltage is applied and the actuator remains flat. Panel (2) illustrates the pin in the up position, resulting from actuator deformation under applied voltage. This bending motion pushes the pin upward, ensuring fast and precise displacement. The actuation mechanism combines high accuracy with low energy consumption, making the module well suited for portable Braille displays. In turn, in the coil module (Figure 3b), the operation sequence is supported by a servo mechanism working together with electromagnetic coils. Panel (1) illustrates the initial position of the pin in the down state. Panel (2) shows the activation of the servo mechanism, which initiates the upward motion of the pin. Panel (3) presents the pin held in the up position, stabilized by the electromagnetic coil acting as a locking element. Finally, panel (4) illustrates the release and return of the pin to the down state once the current is switched off. The servo provides the mechanical motion, while the coils ensure precise locking and unlocking of the pin, combining established electromagnetic principles with a compact actuation system.

### 2.2. Methodology of Performed Tests

Performed analyses included determining the impact of changing temperature and humidity conditions on the functionality and operational stability of both Braille modules, i.e., the coil module and the piezoelectric one. Before the modules were placed in the climate chamber, both modules were mounted on a specially designed frame, which made it possible to test both modules, together with their control systems, at the same time. The frame was designed to provide structural stability and proper working conditions for these modules.

Once mounted on the frame, the device was enclosed in a foamed PVC panel serving as a protective cover, while uniform thermal conditions during testing were maintained by the climate chamber itself. Moreover, this design mimicked the conditions found when using several modules stacked side by side in a finished device. This, in turn, made it possible to consider the thermal interactions of adjacent components. The enclosure used also protected against the direct effects of changing conditions in the climate chamber on the modules, such as rapid temperature fluctuations or sudden changes in humidity, which could lead to uncontrolled effects. In addition, a protective membrane was also used to protect the modules from possible water leakage along the pins. The prepared structure (the frame with modules, housing, and protective membrane) was then placed in a climate chamber. The connection of the power supply and control signals was realized through the grommets of the chamber, which allowed the continuity of control and power supply during testing.

**Figure 4:**
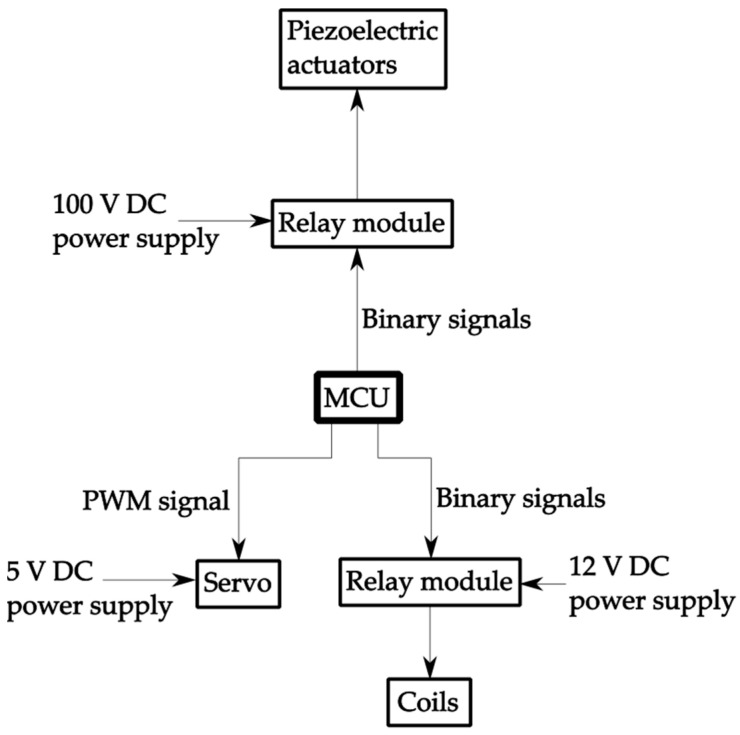
A schematic diagram of the testing setup used to evaluate the performance of the Braille modules.

Below, a schematic diagram of the testing setup is presented (Figure 4). It illustrates the working principle of the system, including interconnections between the power supplies, control modules, servo, and actuators, ensuring a clear overview of the experimental arrangement.

As shown in Figure 4, the testing setup was controlled by a microcontroller unit (MCU), which managed the operation of both piezoelectric and coil-based modules via relay modules and a servo mechanism. Independent power supplies provided 100 V DC for the piezoelectric actuators, 12 V DC for the coils, and 5 V DC for the servo. The MCU generated both binary and PWM control signals, ensuring synchronized and repeatable actuation during testing cycles. This configuration allowed stable and precise operation throughout the experiments.

The main objective of this research was to verify which module performs better under varying conditions as well as to identify potential mechanical problems, which are crucial in terms of their future application. The operation of both modules was verified within the temperature range from −30 °C to +50 °C, while operational effectivity was evaluated every 5 °C. At each of the tested temperatures, 1000 test cycles were performed; one cycle was considered to be the full movement of the module’s pins—from their extension to reaching the end position, and then returning to the initial position. The effective operation criterion was defined as the correct completion of this cycle; i.e., the pin had to fully extend to the active position (pin up) and subsequently return to the resting position (pin down) without jamming, incomplete displacement, or other disturbances. During the testing of the modules, an operating frequency of 1 Hz was used; i.e., each cycle lasted exactly one second. As a result, the modules were subjected to cyclic loads at a constant, moderate rate, which made it possible to closely observe their performance and detect potential interferences.

Importantly, the correct operation of the module in a given cycle was determined as the situation in which the pins correctly returned to their initial position after full extension. A correct cycle, therefore, meant that the movement of the pins proceeded without interference, and the module mechanism operated stably and as intended. Failure to operate correctly was defined as the occurrence of any irregularities in pin movement, as follows:

- self-jamming of the pins in the end position or during return;
- failure to fully return to the initial position, or any interference with movement due to mechanical or thermal problems of the module.

This classification made it possible to accurately assess the performance of the two modules at different temperatures and clearly identify under what conditions they may lose full functionality.

The temperature and operation of the modules were monitored through the viewfinder and the front panel of the climate chamber. The power supply to the modules was provided by cables routed through grommets.

Investigations of the effective operation of both modules were conducted in a climate chamber ESPEC PSL 2J (EMIN Myanmar Co., Ltd., Yangon, Myanmar) working within temperature conditions from −70 °C to 100 °C and relative humidity within the 20–98% range.

## 3. Results

Table 1 demonstrates results of performed tests.

Test results clearly demonstrate differences in the stability and operation of the coil and piezoelectric modules as a function of temperature. The piezoelectric module showed consistent performance over the entire tested temperature range, i.e., from –30 °C to +50 °C. Regardless of the ambient conditions, it did not show any issues such as self-jamming, overheating, or other operational disturbances. Notably, the piezoelectric actuators did not heat up beyond the surrounding temperature during operation, indicating minimal internal energy dissipation.

In contrast, the coil module, while operating stably at temperatures up to +20 °C, began to exhibit problems at higher temperatures. From +20 °C upwards, the module became increasingly unstable—self-jamming was observed, along with significant surface heating. In fact, even with ambient temperatures only slightly above 20 °C, the internal temperature of the coils increased by several tens of degrees Celsius due to pronounced self-heating. This accumulation of heat likely contributed to further mechanical and electrical malfunctions.

## 4. Discussion

**Figure 5:**
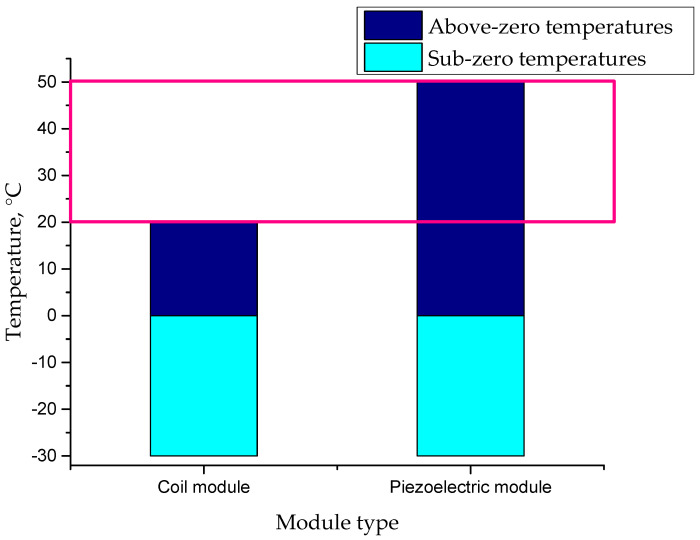
Temperature ranges for coil and piezoelectric modules, showing distinct sub-zero and above-zero ranges. The pink box indicates the temperature interval where the coil module ceases to operate, while the piezoelectric module remains functional.

As can be concluded based on results presented in Figure 5, the piezoelectric module demonstrated better stability and functionality within the tested conditions. The temperature ranges for the stable operation of both modules are schematically presented in the figure below.

It can be observed in Figure 5 that the coil module operated reliably within the temperature range from −30 °C to 20 °C, whereas the piezoelectric module functioned stably across a broader range, from −30 °C to 50 °C. Notably, in the temperature range from 20 °C to 50 °C, the piezoelectric module continued to operate effectively, while the coil module experienced instability and ceased reliable function (this unique operational temperature range is indicated by a pink frame). This demonstrates the piezoelectric module’s superior adaptability to higher temperatures. Hence, it can be stated that piezoelectric technology is well suited to varying environmental conditions, making it potentially more suitable for applications requiring high reliability under varying temperature conditions. Problems with the coil module at higher temperatures can result from several factors arising from its design and operating principle. Firstly, high temperatures can cause thermal expansion of the metal components of a coil module. When using closely connected components, thermal expansion can lead to increased friction and, as a result, the jamming of moving parts. This phenomenon can also affect the geometry of pins, causing blockage or difficulty in moving them.

Additionally, the coil module uses lubricants to improve pin mobility. Nonetheless, at higher temperatures the properties of applied lubricants can change. For example, a reduction in their viscosity can occur, which reduces their effectiveness. Moreover, lower viscosity of lubricants can lead to less effective adhesion between lubricant molecules and the metal surfaces of module components, such as pins and coils. This has been clearly demonstrated in [26]. High adhesion is desirable because it allows the lubricant to adhere to these surfaces, forming a durable protective layer that minimizes friction and enables smooth movement of components. However, at higher temperatures, this adhesion can weaken, making the lubricant less stable on the metal surface. This, in turn, can result in obtaining a thin or irregular layer of lubricant; thus, it can more easily “escape” from the surface, such that it does not form an even protective layer. As a result, there is no adequate lubricant layer, which leads to increased friction between moving parts, making it more difficult to move them. Additionally, there is an increased risk of self-jamming without a proper lubricating layer: parts can jam, and this can especially occur during the mechanical contact under load.

Moreover, electromagnets are applied in the coil module to move the pins, which requires current to flow through their windings. As the coils heat up during operation, the resistance of the conductive material increases. Under constant-voltage drive, this rise in resistance reduces the current, and as Joule heating is proportional to the square of the current and only linearly dependent on the resistance, the system naturally reaches a thermal equilibrium. However, the temperature at this equilibrium can still be high enough to reduce the magnetic force, making smooth pin movement more difficult and increasing the risk of self-jamming. In the studied design, the coils are driven in a pulsed (duty-cycled) mode rather than continuously, which helps limit average heating, but does not eliminate it. Further lowering the supply voltage would reduce the temperature, but would also decrease the available force below the level required for reliable actuation.

To further explain differences in thermal behavior between these two actuator types, it is worth considering their respective equivalent electrical models. The piezoelectric actuator is typically represented as a series RLC circuit, with the series capacitance playing a dominant role. This capacitance blocks direct current (DC), which means that electric current flows only during transient states, such as voltage switching. In steady-state operation, the current is practically zero, and the resulting Joule heating is minimal. Therefore, the self-heating effect in piezoelectric modules is negligible, as energy dissipation occurs only briefly and does not accumulate over time. In contrast, the coil actuator can be modeled as a series RL circuit, which allows significant DC current to flow through the coil once the initial transient has subsided. This results in continuous power dissipation in the form of Joule heating, according to the following Equation (1):

(1) $$P=R\;\cdot \;I^{2}$$

In the studied system, each pin is controlled by two coils—one for locking and one for unlocking—supplied alternately with DC voltage. This configuration effectively turns the module into a steady source of heat during operation. Accumulated thermal energy contributes to an increased surface temperature and destabilizes mechanical operation, particularly under elevated ambient conditions. This circuit-level analysis confirms that the piezoelectric actuator exhibits lower heat generation and maintains stable operational parameters over time.

## 5. Conclusions

- The piezoelectric module demonstrated stable operation over a wider temperature range compared to the coil module, making it more versatile in applications requiring thermal resistance.
- The coil module showed reduced performance at elevated temperatures, with poorer precision and a tendency to self-jam above a certain threshold. Although coil resistance increased with temperature, under constant-voltage supply this led to thermal stabilization rather than runaway heating. The main issue was insufficient heat dissipation from the compact, enclosed coils, causing high operating temperatures, low magnetic force, and deformation of resin-printed parts. Neodymium magnets further limited the thermal tolerance due to their low maximum operating temperature. In contrast, the piezoelectric variant did not self-heat, operated at higher switching speeds, and consumed less power, though it required high voltage (~200 V) and generated lower force, making sealing more challenging.
- These modules’ performance under varying temperature conditions is crucial in the context of their potential applications, especially in environments with wide temperature fluctuations. The piezoelectric module’s better performance at higher temperatures indicates its greater potential for applications requiring reliability and precision under harsh operating conditions.

## Figures and Tables

**Table 1:** Results of tests of the effective operation of modules at various temperature conditions.

Temperature (°C)	Piezoelectric Module	Coil Module
−30	working	working
−25	working	working
−20	working	working
−15	working	working
−10	working	working
−5	working	working
0	working	working
5	working	working
10	working	working
15	working	working
20	working	working
25	working	not working
30	working	not working
35	working	not working
40	working	not working
45	working	not working
50	working	not working

## Data Availability

Data are contained within this article.
